# Apps to improve diet, physical activity and sedentary behaviour in children and adolescents: a review of quality, features and behaviour change techniques

**DOI:** 10.1186/s12966-017-0538-3

**Published:** 2017-06-24

**Authors:** Stephanie Schoeppe, Stephanie Alley, Amanda L. Rebar, Melanie Hayman, Nicola A. Bray, Wendy Van Lippevelde, Jens-Peter Gnam, Philip Bachert, Artur Direito, Corneel Vandelanotte

**Affiliations:** 1 School of Health, Medical and Applied Sciences, Physical Activity Research Group, Central Queensland University, Bruce Highway, Rockhampton, QLD 4702 Australia; 2 Department of Public Health, Ghent University, Ghent, Belgium; 3 Karlsruhe Institute of Technology, Institute of Sports und Sports Science, Karlsruhe, Germany; 4 The University of Auckland, National Institute for Health Innovation, Auckland, New Zealand

**Keywords:** Mobile health (mHealth), Smartphone, Applications, MARS, Behaviour change techniques, Diet, Physical activity, Sedentary behavior, Children, Adolescents

## Abstract

**Background:**

The number of commercial apps to improve health behaviours in children is growing rapidly. While this provides opportunities for promoting health, the content and quality of apps targeting children and adolescents is largely unexplored. This review systematically evaluated the content and quality of apps to improve diet, physical activity and sedentary behaviour in children and adolescents, and examined relationships of app quality ratings with number of app features and behaviour change techniques (BCTs) used.

**Methods:**

Systematic literature searches were conducted in iTunes and Google Play stores between May–November 2016. Apps were included if they targeted children or adolescents, focused on improving diet, physical activity and/or sedentary behaviour, had a user rating of at least 4+ based on at least 20 ratings, and were available in English. App inclusion, downloading and user-testing for quality assessment and content analysis were conducted independently by two reviewers. Spearman correlations were used to examine relationships between app quality, and number of technical app features and BCTs included.

**Results:**

Twenty-five apps were included targeting diet (*n* = 12), physical activity (*n* = 18) and sedentary behaviour (*n* = 7). On a 5-point Mobile App Rating Scale (MARS), overall app quality was moderate (total MARS score: 3.6). Functionality was the highest scoring domain (mean: 4.1, SD: 0.6), followed by aesthetics (mean: 3.8, SD: 0.8), and lower scoring for engagement (mean: 3.6, SD: 0.7) and information quality (mean: 2.8, SD: 0.8). On average, 6 BCTs were identified per app (range: 1–14); the most frequently used BCTs were providing ‘instructions’ (*n* = 19), ‘general encouragement’ (*n* = 18), ‘contingent rewards’ (*n* = 17), and ‘feedback on performance’ (*n* = 13). App quality ratings correlated positively with numbers of technical app features (rho = 0.42, *p* < 0.05) and BCTs included (rho = 0.54, *p* < 0.01).

**Conclusions:**

Popular commercial apps to improve diet, physical activity and sedentary behaviour in children and adolescents had moderate quality overall, scored higher in terms of functionality. Most apps incorporated some BCTs and higher quality apps included more app features and BCTs. Future app development should identify factors that promote users’ app engagement, be tailored to specific population groups, and be informed by health behaviour theories.

**Electronic supplementary material:**

The online version of this article (doi:10.1186/s12966-017-0538-3) contains supplementary material, which is available to authorized users.

## Background

Unhealthy diet, physical inactivity and sedentary behaviour are highly prevalent health risk factors in children and adolescents [1]. These health behaviours are known to track from childhood into adulthood [2, 3], and contribute to high rates of childhood overweight/obesity, and an increased prevalence of type 2 diabetes and metabolic syndrome in adolescence [4]. Given the scale of the problem – worldwide over 41 million children under 5 years of age are already overweight or obese [5] – population-based interventions that can reach large numbers of children and adolescents easily and at low cost are needed. Smartphone applications are widely used (there are over 2.1 billion smartphone users worldwide) [6] and can reach large numbers of children in real life situations where they live, learn and play.

Smartphones and tablets, including the software applications (apps) that run on these devices, have become an integral part of children and adolescents’ lives with large increases in usage rates since their introduction in 2007 [6, 7]. For example, 73% of American, 74% of European and 80% of Australian adolescents regularly use a smartphone [8–10]. With the growth in mobile technologies came the development and popularity of numerous health and fitness apps that can provide behavioural interventions in large population groups [11]. Given the proliferation of apps, it is worthwhile to investigate their potential for promoting healthy lifestyle behaviours in children and adolescents. The appeal of commercial apps to provide health information ‘on the go’ has motivated researchers to utilise commercial apps for behavioural interventions that incorporate proven health behaviour changes techniques (BCTs) such self-monitoring, real-time feedback, social support, and rewards [12].

Despite the potential of apps for pediatric health behaviour change interventions, the behaviour change content and quality of apps specifically targeted to children and adolescents is largely unexplored. Several systematic reviews [11, 13–18] have examined the content of apps to promote healthy lifestyle behaviours in adults or the general population, and their results showed that most apps included self-monitoring, goal-setting, instructions on how to perform a health behaviour and feedback on performance. Only one systematic review [17] has evaluated the content of commercial health and fitness apps targeted to children and adolescents, and its findings showed that apps incorporated gamification elements and goal-setting but lacked concrete expert recommendations about healthy lifestyle behaviours. However, this review [17] focused on weight loss and addressed diet and physical activity, but not sedentary behaviours. In fact, many previous reviews of apps targeting adults [11, 14, 15, 19] have mainly focused on apps to promote physical activity. As such, little is known about the potential of using apps to improve diet and sedentary behaviour. Moreover, most previous reviews examining the content of apps limited their searches to apps available in iTunes only [14, 16–18, 20] and solely to apps provided in the ‘health and fitness’ category [13–16, 18, 20, 21]. These selection criteria help reduce the number of apps eligible for assessment which facilitates a feasible review process. However, many widely used and popular apps may be missed which limits the conclusions that can be drawn from the review findings. Finally, most previous reviews [11, 13–17] have solely assessed the incorporation of established BCTs in apps. To our knowledge, only one review targeted to adults [22] has evaluated app quality criteria in relation to engagement, functionality, aesthetics and information quality of apps. These have shown to be important factors that influence user’s engagement with an app [12, 23], and ultimately, the effectiveness of app-based health behaviour interventions [24].

To address the highlighted gaps in the literature, this review aimed to 1) systematically evaluate the content and quality of popular apps to improve diet, physical activity and sedentary behaviour in children and adolescents, and to 2) examine the relationships between app quality and number of app features and BCTs incorporated in the app.

## Methods

### Search strategy

Systematic searches were conducted in the Australian Apple iTunes and Google Play stores between May and November 2016 (see Additional file 1). Apps were identified using the following search terms: physical activity, physical fitness, exercise, sport, walk, sedentary behaviour, sitting, inactive, screen time, diet, nutrition, healthy eating, fruit, vegetable, snack, soft drink and carbonated beverages. The search terms were entered separately in the iTunes and Google Play databases with and without specified search categories including education, food and drink, health and fitness, sports, lifestyle, games, kids and family. For search terms producing over 1000 apps, the title and description of several sets of 100 apps were screened until no more apps were identified in a set of 100 apps shown on the app store’s webpage. In addition to the search in app stores, apps were identified by reviewing the apps included in similar reviews [16, 20].

### Inclusion criteria and selection process

This review included apps that were available in iTunes, Google Play or both stores. Apps were included if they (i) targeted children or adolescents, (ii) focused on at least one of the following energy-balance behaviours: diet, physical activity and sedentary behaviour, (iii) were available in English and (iv) had a user rating of at least 4+ (scale range: 1–5) based on at least 20 ratings in either the iTunes or Google Play store (as done elsewhere [22]). The reason for applying a user rating threshold as inclusion criterion was to take app popularity into account. Apps targeting children or adolescents were considered, if the app description published in the iTunes and Google play stores specified targeted child/adolescent ages, or if at least two reviewers (SS, NB) who independently reviewed the app for inclusion considered it suitable for children or adolescents. Disagreement was resolved by discussion and consensus with a third reviewer (CV). In addition, apps could (but were not required) target families, be administered in combination with or without an external device (e.g., physical activity tracker), be a serious game, and be a free, paid or freemium (i.e., free app with limited functionality which is unlocked by purchasing the full version) app. Apps targeting adults and addressing other health behaviours were excluded from the review. App selection and assessment were undertaken between June and November 2016. As per best practice for systematic reviews [25], two reviewers (SS, NB) independently reviewed the titles and descriptions of each identified app for inclusion in the review. Disagreement was resolved by discussion and consensus with a third reviewer (CV). Subsequently, eligible apps were downloaded to a smartphone (iphone or Android), user-tested and assessed for content including technical app features, incorporation of BCTs and quality criteria. If an app was available in both iTunes and Google Play, either version could be utilised for the user testing; the choice was determined by the smartphone (iPhone or Android) used by the reviewer. Further, if an app had a free version and a paid version, the free version was downloaded first. If the paid version had extra features not available in the free version, it was also downloaded and evaluated.

### Data extraction

Data extraction was conducted using a standardised scale developed specifically for this review (see Additional file 2); similar to those used in previous app reviews [16, 17, 22]. For all included apps, data were extracted for app name, developer, store (Apple iTunes, Google Play), cost (free, freemium, paid), average user rating (at least 4+), number of user ratings (at least 20), target group (i.e., children, adolescents), target age range (if reported by app developers), health behaviour (diet, physical activity, sedentary behaviour), app type (e.g., educational, exergame, serious game), app functionalities (i.e., password required, inbuilt accelerometer, GPS, educational information, alignment with guidelines for diet, physical activity and/or sedentary behaviour, social networking option, push notifications, reminders, awards/rewards, gamification), combination with other devices (e.g., wearable physical activity tracker), and BCTs incorporated. An app included gamification if it was designed as a game (e.g., exergame, serious game) or included gamification tools such as badges/medals/coins/points, competition, achievement, self-expression and leaderboards to keep users engaged. The presence or absence of BCTs for improving diet, physical activity and/or sedentary behaviour was assessed using the taxonomy of BCTs developed by Abraham and Michie [26]. A dichotomous score of “0” absent or “1” present was applied for each of the 26 BCTs resulting in a total score of 0–26 (see Additional file 3). This approach has been applied in similar app reviews and content analyses [11, 16]. The app testing and data extraction was conducted independently by two reviewers (all authors contributed), with any disagreement being resolved by discussion and/or consultation of a third reviewer (SS).

### Quality assessment

App quality was assessed using the Mobile App Rating Scale (MARS) [27]; this approach has been used in a similar app review [22]. The MARS consists of 19 items grouped in four domains: 1) engagement (entertainment, interest, customisation, interactivity, and target group); 2) functionality (performance, ease of use, navigation, gestural design); 3) aesthetics (layout, graphics, visual appeal); 4) information quality (accuracy of app description, goals, quality and quantity of information, visual information, credibility, evidence base). All items are measured on a 5-point scale (1 = inadequate to 5 = excellent). A score for each domain is computed as the mean of the items in that domain; an overall score is computed as an average across the domains [27]. The app quality assessment was conducted independently by two reviewers (all authors contributed). Disagreement between the two reviewers by 1-point was resolved by taking the mean of the two ratings. Disagreement by more than 1-point was resolved by discussion and/or consensus with a third reviewer (see Additional file 4).

### Statistical analyses

Frequencies (numbers, percentages) of each of the 26 BCTs included in the apps were calculated. Krippendorff’s alpha (Kalpha) was used to evaluate interrater reliability for the app quality assessment and the presence of BCTs in the apps [28]. Spearman correlations were used to examine the relationships between app quality, number of technical app features and number of BCTs incorporated in the apps. All statistical analyses were conducted using IBM SPSS Statistics version 22.0 with significance levels set at *p* < 0.05.

## Results

### App selection

**Fig. 1:**
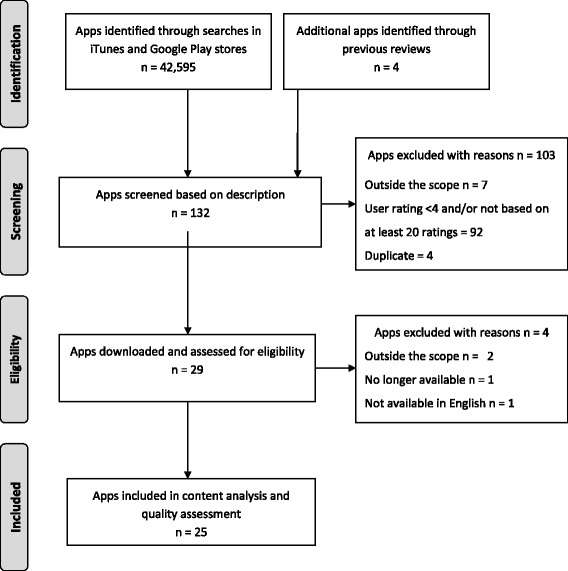
Flowchart of app selection process

A flowchart of the app selection process is presented in Fig. 1. A total of 42,599 apps were identified and screened in the Apple iTunes and Google Play stores. Of these, 132 apps were further screened by description, and 29 apps were considered eligible for inclusion and downloaded for testing. After testing and confirming eligibility, 25 apps targeting diet, physical activity and/or sedentary behaviour in children and/or adolescents were included in the content analysis and quality assessment.

### App characteristics

Characteristics of the apps included in this review are presented in Table 1. Three apps were identified in iTunes, four apps in Google Play and 18 apps in both stores. Most apps were freely available (*n* = 15); fewer apps were freemium (*n* = 6) or paid (*n* = 4). The targeted health behaviours were diet (*n* = 12), physical activity (*n* = 18) and sedentary behaviour (*n* = 7), whereas 11 apps targeted more than one health behaviour (*n* = 4 diet and physical activity, *n* = 5 physical activity and sedentary behaviour, *n* = 1 diet and sedentary behaviour, *n* = 1 diet, physical activity and sedentary behaviour). Seventeen apps were stand-alone apps and 8 apps operated in combination with websites (*n* = 4) and/or wearable physical activity trackers (*n* = 8). Most apps incorporated gamification (*n* = 21), rewards/awards (*n* = 17), educational information (*n* = 17) and social networking options (*n* = 15). Fewer apps used the smartphone’s inbuilt accelerometer (*n* = 9), push notifications (*n* = 8), reminders (*n* = 6), required a password prior to usage (*n* = 5), included a GPS function (*n* = 5) or were aligned with dietary, physical activity or sedentary behaviour guidelines (*n* = 6). Apps targeted children and adolescents with age ranging from 2 to 18 years.

**Table 1:** Descriptive data of the included apps (*n* = 25)

	*N* (%)	M (SD)	Md (IQR)	Range (min-max)
Store				
iTunes	3 (12)	-	-	-
Google Play	4 (16)	-	-	-
iTunes and Google Play	18 (72)	-	-	-
Cost				
Free	15 (60)	-	-	-
Paid (costs in AUD)	4 (16)	3.49	-	6.50 (1.49–7.99)
Freemium	6 (24)	-	-	-
User rating				
Average rating (4+)	25 (100)	4.28 (0.26)	4.30 (0.50)	0.80 (4.00–4.80)
Average number of user ratings (count)	25 (100)	279,611.33 (1,315,200.00)	190.00 (3418)	6,451,103 (22–6,451,125)
Health behaviour				
Diet	12 (48)	-	-	-
Physical activity	18 (72)	-	-	-
Sedentary behaviour	7 (28)	-	-	-
App type				
Educational	17 (68)	-	-	-
Game (serious game, exergame)	13 (52)	-	-	-
Other	5 (20)	-	-	-
Number of app features (1–10)		4.32 (1.93)	4.00 (3.00)	7.00 (1.00–8.00)
App features specified				
Password required	5 (20)	-	-	-
Inbuilt accelerometer	9 (36)	-	-	-
GPS	5 (20)	-	-	-
Educational information	17 (68)	-	-	-
Alignment with guidelines	6 (24)	-	-	-
Social networking option	15 (60)	-	-	-
Push notifications	8 (32)	-	-	-
Reminders	6 (24)	-	-	-
Awards/rewards	17 (68)	-	-	-
Gamification	21 (84)	-	-	-
Combination with other devices				
None	17 (68)	-	-	-
Wearable tracker	8 (32)	-	-	-
Website	4 (16)	-	-	-
Number of BCTs	25 (100)	6.12 (3.38)	6.00 (6)	14 (0–14)
MARS app quality ratings (1–5)				
Engagement score	25 (100)	3.57 (0.73)	3.70 (1.05)	2.70 (2.00–4.70)
Functionality score	25 (100)	4.10 (0.58)	4.00 (1.00)	1.90 (3.00–4.90)
Aesthetics score	25 (100)	3.81 (0.81)	3.80 (1.35)	2.80 (2.00–4.80)
Information quality score	25 (100)	2.79 (0.84)	2.90 (1.20)	3.30 (0.90–4.20)
Total score	25 (100)	3.57 (0.60)	3.70 (0.85)	2.00 (2.40–4.40)

*Abbreviations: App* application, *BCTs* behaviour change techniques, *MARS* mobile app rating scale, *GPS* global positioning system, *AUD* Australian Dollar

### Presence of behaviour change techniques

**Fig. 2:**
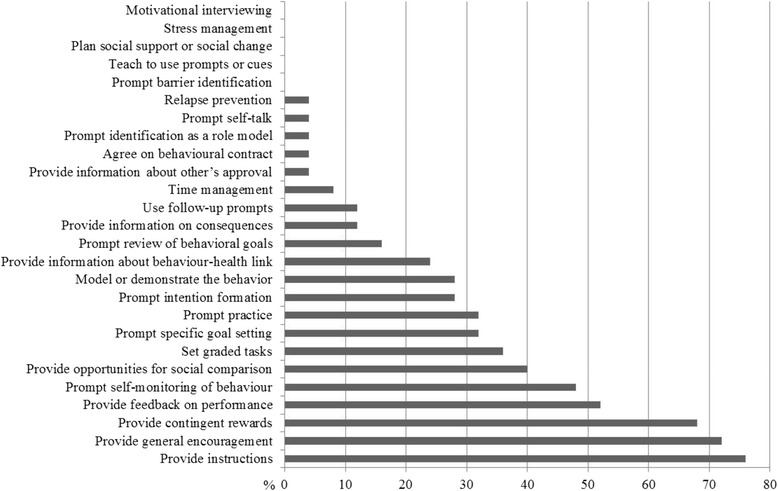
Presence of behaviour change techniques in the apps

The number and types of BCTs included in the apps are presented in Fig. 2 and Additional file 3. Inter-rater reliability for evaluating the presence of BCTs in the apps was good, measured by Krippendorff’s alpha and percent agreement between reviewer 1 and 2 (Kalpha = 0.71, percent agreement = 87%). Overall, 24 out of the 25 apps incorporated some BCTs. Commonly included BCTs were ‘Provide instructions’ (*n* = 19 out of 26, 76%), ‘Provide general encouragement’ (*n* = 18 out of 26, 72%), ‘Provide contingent rewards’ (*n* = 17 out of 26, 68%), ‘Provide feedback on performance’ (*n* = 13 out of 26, 52%), ‘Prompt self-monitoring of behaviour’ (*n* = 12 out of 26, 48%), and ‘Provide opportunities for social comparison’(*n* = 10 out of 26, 40%). The average number of BCTs per app was 6, ranging from 0 to 14. Apps with the highest number of BCTs included were Zombies Run 5 K (*n* = 14), Kurbo (*n* = 12), iBitz (*n* = 10) and NFL Play 60 (*n* = 10).

### App quality

A detailed summary of quality assessment of the included apps is presented in the Additional file 4. Inter-rater reliability for app quality was acceptable (Kalpha = 0.73). The average total MARS score was 3.6 out of 5 with a range of 2.4–4.4. Functionality was the highest scoring domain (mean: 4.1, SD: 0.6), followed by aesthetics (mean: 3.8, SD: 0.8), engagement (mean: 3.6, SD: 0.7) and information quality (mean: 2.8, SD: 0.8).

### Relationships between app quality, app features and behaviour change techniques

Spearman correlation between app quality, number of app features and number of BCTs are presented in Table 2. Number of included BCTs was positively associated with the total MARS score (rho = 0.54, *p* < 0.01), and the MARS engagement score (rho = 0.74, *p* < 0.01) and information quality score (rho = 0.48, *p* < 0.05). Functionality and aesthetics were not significantly correlated with number of BCTs. Number of app features was positively associated with the total MARS score (rho = 0.42, *p* < 0.05), and the MARS engagement score (rho = 0.70, *p* < 0.01) but not with the other MARS sub-scores. Further, number of app features were positively associated with number of BCTs (rho = 0.77, *p* < 0.01).

**Table 2:** Correlations between app quality, number of app features and BCTs

	Number of BCTs	Number of app features
MARS engagement score	0.74**	0.70**
MARS functionality score	0.04	−0.04
MARS aesthetics score	0.29	0.21
MARS information quality score	0.48*	0.35
MARS total score	0.54**	0.42*
Number of app features	0.77**	1.00

*Abbreviations: MARS* mobile app rating scale, *BCTs* behaviour change techniques

**p < 0.01

*p < 0.05

## Discussion

This review assessed the content and quality of popular (4+ user rating) commercial apps to improve diet, physical activity and sedentary behaviour in children and adolescents. Furthermore, relationships between app quality, technical app features and BCTs used in the apps were examined. Overall, app quality was moderate but scored higher in terms of functionality. On average, the identified apps included four app features and six BCTs. Apps with higher (MARS) quality tended to incorporate more app features and BCTs.

Generally, we noticed that fewer apps were available in the app stores that specifically targeted children or adolescents, compared to apps targeting adults. This may partially explain the lower number of apps included in this review compared to those included in previous reviews, which mostly focused on apps targeting adults or the general population [11, 13–18]. App quality was moderate, with the highest scoring domain being functionality, followed by aesthetics and engagement, and the lowest scoring domain being information quality. This suggests that developers of commercial health and fitness apps have responded to users’ preference for functional and easy to use apps [12, 23, 29]. The low MARS score for information quality, however, reinforces the need for apps with evidence-based content [22]. The low MARS score for engagement indicates another domain for improvement in future app development. Better understanding of factors that improve children and adolescents’ engagement with an app is needed. App and internet usage data [10] has shown that children and adolescents primarily use photos and videos, download music, play games and engage in social networking, all of which demonstrates the desire for entertainment and social connectedness with peers. These and other user engagement factors are important considerations in future app development, especially since engagement with an app is positively associated with their effectiveness to improve health behaviours [30, 31].

There was substantial variation in the number of BCTs incorporated in an app (range 0–14), with an average of six BCTs per app out of the 26 BCT taxonomy developed by Abraham and Michie [26]. This is consistent with previous reviews of apps targeting adults [11, 13, 16] where the average number of BCTs used in apps ranged between 4 and 8. Similarly to these reviews [11, 13, 16], our findings demonstrate that commercial apps targeting health behaviours have ignored many BCTs associated with intervention effectiveness [32]. Reviews of health behaviour interventions using websites have shown that interventions including more BCTs are more effective [33, 34]. This may also apply to app-based interventions. However, it remains unclear what the optimal number and combination of BCTs is to increase the effectiveness of apps to improve diet, physical activity and sedentary behaviour. It is also possible that user engagement and app effectiveness decline when too many BCTs are incorporated. In this review, the most frequently used BCTs were ‘Provide instructions’, ‘Provide general encouragement’, ‘Provide contingent rewards’, and ‘Provide feedback on performance’. This is somewhat different to the findings from reviews of apps targeting dietary, physical activity and sedentary behaviours in adults [11, 14, 22], which found goal-setting, self-monitoring and performance feedback to be the most frequently used BCTs. Furthermore, the most frequently used BCTs identified in our app review do not represent the most effective BCTs in children and adolescents which have shown to be social support and modelling in children, and social support, modelling, consequences for behaviour, other’s approval, self-monitoring, intention formation and behavioural contracting in adolescents [35]. This is unfortunate and demonstrates the need for incorporation of more effective behavioural strategies in apps targeting children and adolescents.

Most apps included technical features such as educational information, social networking options, rewards/awards and gamification. The incorporation of social networking options in apps shows that app developers have already addressed young people’s interest in social media such as Facebook and their desire to connect with peers for motivation and support [10]. Fewer apps incorporated an inbuilt monitoring system (e.g., accelerometer, GPS), push notifications and reminders; and very few apps were aligned with established dietary, physical activity or sedentary behaviour guidelines. These technical features could be improved in future app designs as focus group data [12] has shown that young people value health behaviour apps that enable self-monitoring, provide advice on how to change behaviour and include positively framed alerts/reminders (though not too frequent).

There was a positive relationship between overall app quality and number of technical app features and BCTs incorporated in the app. In particular, the MARS score engagement correlated highly with number of app features and BCTs. This finding is consistent with those found in a review of apps targeted to adults [22] and suggests that offering app features to support specific BCTs may improve the perceived functionality, aesthetics and engagement of the app and lead to repeated use [22]. In this context, it worth noting though that the BCT coding (i.e. BCT present yes/no) has its limitations in that it does not capture how well a BCT is operationalised in the app. For example, if a BCT is not well implemented, it could undermine its purpose (e.g. modelling will not work if the target population does not identify with the role model), and therefore affect user engagement. Nonetheless, the positive relationship between number of BCTs and app quality observed in this app review suggests that the incorporation of multiple BCTs tailored to specific child and adolescent populations is vital for its attractiveness to users.

A strength of this app review is that it used a taxonomy of proven BCTs [26] to assess the content of apps, and quality was further evaluated using the MARS instrument [27], which was specifically developed to assess the design and usability of smartphone apps including user engagement, functionality, aesthetics, and information quality. Moreover, this review focused on popular apps with a user rating of at least 4+ (scale range: 1–5) based on at least 20 ratings, in an attempt to learn how to improve app designs and users’ engagement with apps. Such in-depth assessment of app quality and popularity has rarely been undertaken in previous reviews in this area [22]. Moreover, the search strategy was comprehensive, and app selection, data extraction, content analysis and quality assessment were completed by two independent reviewers, as is standard practice for high quality systematic reviews [25]. These procedures reduce the risk of inaccuracy of the reviewed data. This review also has several limitations. It was limited to apps addressing diet, physical activity and sedentary behaviour; apps relating to other lifestyle behaviour were not captured in this review. Few apps identified in this review focused on sedentary behaviour which makes it more difficult to draw conclusions on the content, quality and usability of apps targeting sedentary behaviour, as opposed to those targeting dietary and physical activity behaviours. Furthermore, the included apps varied widely in terms of quality, with some apps scoring very poorly, thereby reducing the trust that can be placed in their usability, usefulness and potential effectiveness in behavioural interventions. Finally, the possibility of assessment bias should be acknowledged. Although app quality was assessed independently by two researchers it is possible that ratings were subject to individual preference. Moreover, children and adolescents may have rated functionality, engagement, aesthetics and information quality differently compared to adults.

## Recommendations for future app developments

Based on this review, it is recommended that researchers, health professionals and app developers involved in app design:

- Pilot-test several most popular commercially available apps that incorporate diverse BCTs in child and adolescent populations in terms of usability, usefulness and usage. This can inform the development of more effective, high quality and user friendly apps.
- Incorporate more BCTs in the app design and test which combination of BCTs are most appealing and effective in specific child and adolescent populations.
- Design apps for specific child populations (e.g., children, adolescents, girls, boys) in whom usage and adoption of app technology is high.
- Provide educational information and health behaviour change advice informed by evidence-based dietary, physical activity and sedentary behaviour guidelines.
- Compare user’s engagement with stand-alone apps versus those that are used in combination with wearable activity trackers, websites and/or social media.
- Report app usage statistics using objective and self-report measures to examine levels of and reasons for user’s (dis)engagement with the app.
- Identify factors that increase user’s engagement with an app to facilitate long-term health behaviour improvements.
- Foster effective communication and collaboration between health behaviour change researchers and app developers to set clear expectations about the app design and learn from each other.

## Conclusions

Popular (4+ user rating) commercial health and fitness apps to improve diet, physical activity and sedentary behaviour in children and adolescents show moderate quality overall. They score high for functionality, moderate for aesthetics and engagement, and low for quality information. Most apps identified incorporate some BCTs, with the most frequently used BCTs being providing instructions, general encouragement, contingent rewards and performance feedback. Higher quality apps tended to incorporate more technical app features and BCTs. There is still considerable scope to improve the effectiveness of apps to engage users and ultimately improve health behaviours. App development should identify factors that promote user engagement with the app, be tailored to specific population groups, and informed by evidence-based health behaviour guidelines and theories. More formative research is needed to determine the optimal number and combination of app features and BCTs needed to maximise app quality and user engagement.

## Additional files

Additional file 1: Search strategies. (DOCX 15 kb)

Additional file 2: Detailed characteristics of the included apps. (DOCX 103 kb)

Additional file 3: BCT scores and inter-rater reliability. (DOCX 56 kb)

Additional file 4: MARS scores and inter-rater reliability. (DOCX 49 kb)

## Abbreviations

apps: Applications
BCTs: Behaviour change techniques
GPS: Global positioning system
MARS: Mobile app rating scale
