# Supplementary material for: Apps to improve diet, physical activity and sedentary behaviour in children and adolescents: a review of quality, features and behaviour change techniques
Source: Int J Behav Nutr Phys Act. 2017 Jun 24;14:83. doi: 10.1186/s12966-017-0538-3 (PMC5483249; doi:10.1186/s12966-017-0538-3)
Supplement: Supplementary file 1 — Search strategies. (DOCX 15 kb) [file 12966_2017_538_MOESM1_ESM.docx]

**Additional file 1 Search strategies used in the iTunes and Google Play stores**

General search terms and categories used in the searches

| Search terms | physical activity OR walk, OR physical fitness OR exercise OR sport OR sedentary OR sitting OR inactive OR screen time OR diet OR nutrition OR healthy eating OR fruit OR vegetable OR snack OR soft drink OR carbonated beverages |
| --- | --- |
| Search categories | Education OR food & drink OR games OR health & fitness OR lifestyle OR sports |
| Limits | Children, adolescents  Focus is diet, physical activity, sedentary behaviour  Available in English |

**iTunes**

Dates of the search: Between 8 May and 30 November 2016

Total #Hits = 79,086

#Hits by search terms:

physical activity [79], walk [3985], physical fitness [202], exercise [6511], sport [8523] sedentary [13], sitting [185], inactive [8], screen time [39], diet [5519], nutrition [3081], healthy eating [478], fruit [9000], vegetable [981], snack [696], soft drink [281], carbonated beverages [1]

#Hits by search category:

Education [4024] food & drink [340] games [16526] health & fitness [10591] lifestyle [7021] sports [7842] kids [162]

**Google Play**

Dates of the search: Between 8 May and 30 November 2016

Total #Hits = 6,028

#Hits by search terms:

physical activity [100], walk [249], physical fitness [177], exercise [250], sport [236] sedentary [100], sitting [177], inactive [249], screen time [238] diet [249], nutrition [242], healthy eating [100], fruit [248], vegetable [99], snack [99], soft drink [100], carbonated beverages [100]

#Hits by search category:

Education [55] food & drink [400] games [674] health & fitness [731] lifestyle [385] sports [770]
