# Supplementary material for: Apps to improve diet, physical activity and sedentary behaviour in children and adolescents: a review of quality, features and behaviour change techniques
Source: Int J Behav Nutr Phys Act. 2017 Jun 24;14:83. doi: 10.1186/s12966-017-0538-3 (PMC5483249; doi:10.1186/s12966-017-0538-3)
Supplement: Supplementary file 2 — Detailed characteristics of the included apps. (DOCX 103 kb) [file 12966_2017_538_MOESM2_ESM.docx]

Table 1: Characteristics of the apps included in the review

| Name  **Developer**  Store | **Cost**  User rating  **Target group**  **Health behaviour** | **App type**  **App features**  **Combined with other devices** | **Behaviour change technique** |
| --- | --- | --- | --- |
| **Name**  Walkr  **Developer**  Fourdesire  **Store**  iTunes  Google Play | **Cost**  Freemium  **User rating**  Average: 4.5  Number of user ratings: 34,391  **Target group**  Children  Adolescents  **Health behaviour**  Physical activity | **App type**  Exergame  **App features**  n = 6  Password required: no  Inbuilt accelerometer: yes  GPS: no  Educational information: no  Alignment with guidelines: no  Social networking option: yes   - Connects to Facebook (option to share success and compare level with social media friends)   Push notifications: yes   - ‘Your planet finished production,’ ‘Captain, we’ve discovered a new planet’   Reminders: yes   - Reminder that energy is needed   Awards/rewards: yes (‘Wow you generated new energy’ after walking m ‘You have earned your daily reward’, ‘Congratulations, you completed the achievement. 5 coins.’)  Gamification: yes   - ‘Wow, you generated 500 new energy.’; Planets discovered, feeding populations   **Combined with other devices**  Apple watch | **Behaviour change technique**  n = 6  Provides general encouragement  Sets graded tasks  Prompts self-monitoring of behaviour  Provide contingent rewards  Uses follow-up prompts  Provides opportunities for social comparison |
| **Name**  Wokamon – Monster Walk  **Developer**  Noodum Co. Ltd.  **Store**  iTunes  Google Play | **Cost**  Freemium  **User rating**  Average: 4.3  Number of user ratings: 4,344  **Target group**  Young children  Target age range: 2 – 8 years  **Health behaviour**  Physical activity  Sedentary behaviour | **App type**  Exergame  **App features**  n = 5  Password required: no  Inbuilt accelerometer: yes  GPS: yes  Educational information: no  Alignment with guidelines: no  Social networking option: yes   - Connects to Facebook, Twitter, Pinterest to share accomplishments   Push notifications: no  Reminders: no  Awards/rewards: yes   - Calories burned equal food and gold to feed and grow monster pets   Gamification: yes   - Activity hatches Wokaman, provides gems to use within game, and levels them up   **Combination with other devices**  Website, wearable tracker (e.g., Google Fit, Fitbit, UP, Jawbone, Moves, Mi band and LedongLi) | **Behaviour change techniques**  n = 7  Provide instruction  Provides general encouragement  Sets graded tasks  Prompts self-monitoring of behaviour  Provides contingent rewards  Provide feedback on performance  Provides opportunities for social comparison |
| **Name**  Zombies Run 5k Training  **Developer**  Six to Start & Naomi Alderman  **Store**  iTunes  Google Play | **Cost**  Paid  **User rating**  Average: 4.3  Number of user ratings: 3,797  **Popularity**  **Target group**  Adolescents  Age range: 12+ years  **Health behaviour**  Physical activity  Sedentary behaviour | **App type**  Exergame  **App features**  n = 6  Password required: no  Inbuilt accelerometer: yes  GPS: yes  Educational information: yes  Alignment with guidelines: no  Social networking option: yes   - Connects to Facebook, Twitter and ZombieLink to share progress   Push notifications: no  Reminders: no  Awards/rewards: yes   - Verbal praise, each workout unlocks the next, collect medical kits and other rewards along the way to help your community survive.   Gamification: yes   - Training aim is to be able to conquer zombies   **Combination with other devices**  Website, wearable tracker (Fitbit) | **Behaviour change techniques**  n = 14  Provide information about behaviour health link  Prompts intention formation  Provide general encouragement  Sets graded tasks  Provides instructions  Prompts specific goal setting  Prompts review of behavioural goals  Provide feedback on performance  Prompts self-monitoring of behaviour  Provides contingent rewards  Agrees on behavioural contract  Prompts practice  Uses follow-up prompts  Prompts self-talk |
| **Name**  Pokemon Go  **Developer**  Niantic Inc.  **Store**  iTunes  Google Play | **Cost**  Freemium  **User rating**  Average: 4.1  Number of user ratings: 6,451,125  **Target group**  Children  Adolescents  Adults  Age range: 9+ years  **Health behaviour**  Physical activity | **App type**  Exergame  **App features**  n = 5  Password required: no  Inbuilt accelerometer: yes (but only to judge if driving)  GPS: yes  Educational information: no  Alignment with guidelines: no  Social networking option: yes   - Connects to Facebook, Twitter, Youtube and Google Plus   Push notifications: no  Reminders: no  Awards/rewards: yes   - Incubated eggs will hatch and pokemon to collect depending on distance walked or cycled.   Gamification: yes   - Players can collect virtual items at ‘PokeStops’, hatch eggs containing new Pokemon by walking certain distances (usually between 2km and 10km) and compete against other users to capture gyms   **Combination with other devices:**  Apple watch | **Behaviour change techniques**  n = 6  Provide instruction  Provides general encouragement  Prompt intention formation  Prompt specific goal setting  Prompts self-monitoring of behaviour  Provides contingent rewards |
| Name  Sworkit Kids  **Developer**  Nexercise Apps Inc.  Store  Google Play | **Cost**  Free  User rating  4.7  Number of ratings: 3968  **Target group**  Young children  Age group: under 12  **Health behaviour**  Physical activity  Exercise | **App type**  Educational  **App features**  n = 3  Password required: no  Inbuilt accelerometer: no  GPS: no  Educational information: yes   - Instructions on how to perform exercises   Alignment with guidelines: no  Social networking option: no  Push notifications: yes   - To exercise   Reminders: yes   - Training reminders   Awards/rewards: no  Gamification: no  **Combination with other devices**  None | **Behaviour change techniques**  n = 5  Provide general encouragement  Provide instruction  Provide feedback on performance  Set graded tasks  Model or demonstrate the behavior |
| Name  Fitbit  **Developer**  Fitbit Inc.  Store  iTunes  Google Play | **Cost**  Free  User rating  4.5  Ratings: 205,904  **Target group**  General  Adolescents (13-17 years)  **Health behaviour**  Diet  Physical activity  sleep | **App type**  Educational  **App features**  n = 7  Password required: no  Inbuilt accelerometer: yes  GPS: yes  Educational information: yes   - Instructions regarding diet and sleep   Alignment with guidelines: no  Social networking option: yes   - Connects to Facebook   Push notifications: yes   - Goal attainment   Reminders: yes  Awards/rewards: yes   - Dietary goals   Gamification: no  **Combination with other devices**  Website, different wearable tracker | **Behaviour change techniques**  n = 8  Provide general encouragement  Provide instruction  Prompt specific goal setting  Prompt review of behavioral goals  Prompt self-monitoring of behavior  Provide feedback on performance  Provide contingent rewards  Provide opportunities for social  comparison |
| **Name**  Awesome Eats  **Developer**  Whole Kids Foundation (provider) & Fun Machine (developer)  **Store**  iTunes  Google Play | **Cost**  Free  **User rating**  Average: 4.3  Number of user ratings:  2464  **Target group**  Young children  Children  Age range: 4+ years  **Health behaviour**  Diet  Sedentary behaviour | **App type**  Educational  **App features**  n = 4  Password required: no  Inbuilt accelerometer: no  GPS: no  Educational information: yes   - Many tips like ‘eat fruit and vegetables in as many colours as you can to provide a broad range of plant nutrients and antioxidants’ or ‘you can add protein and extra crunch to a salad by topping with nuts or seeds’.   Alignment with guidelines: no  Social networking option: yes   - Connects to Facebook for Likes and challenge with other users.   Push notifications: no  Reminders: no  Awards/rewards: yes   - When you sort the fruit and vegetables into correct basket, you are awarded points and badges   Gamification: yes   - Rewards   **Combination with other devices**  None | **Behaviour change techniques**  n = 5  Provide information about behaviour-health  link  Provide general encouragement  Provide contingent rewards  Prompt intention formation  Provide instruction |
| **Name**  RunBit  **Developer**  QuidBit AB  **Store**  iTunes  Google Play | **Cost**  Freemium  **User rating**  Average: 4.0  Number of user ratings:  195  **Target group**  Young children  Children  Adolescents  Adults  Age range: 4+ years  **Health behaviour**  Physical activity  Sedentary behaviour | **App type**  Exergame  **App features**  n = 5  Password required: no  Inbuilt accelerometer: yes  GPS: yes  Educational information: no  Alignment with guidelines: no  Social networking option: yes   - Connects to Facebook, Google Plus, Instagram, Linkedin and Twitter.   Push notifications: no  Reminders: no  Awards/rewards: yes   - Collect stars, collect points for escaping monsters and chasing animals   Gamification: yes   - Collect stars, collect points for escaping monsters and chasing animals   **Combination with other devices**  Apple watch, other apps (e.g., (endomondo, nike+fuel), wearable tracker (e.g. endomondo, nike plus) | **Behaviour change techniques**  n = 3  Provide feedback on performance  Provide contingent rewards  Provide opportunities for social comparison |
| **Name**  Dungeon Runner  **Developer**  Six to Start  **Store**  iTunes | **Cost**  Free  **User rating**  4+  Number of user ratings: 128  **Target group**  Adolescents  Age range: 12+ years  **Health behaviour**  Physical activity | **App type**  Educational  **App features**  n = 3  Password required: no  Inbuilt accelerometer: yes  GPS: no  Educational information: no  Alignment with guidelines: no  Social networking option: yes   - Connects to social media (option to share level completion).   Push notifications: no  Reminders: no  Awards/rewards: no  Gamification: yes   - Level 1 completed, 183m, 1:44 minutes, 5.1 calories and 4 kills   **Combined with other devices**  None | **Behaviour change technique**  n = 2  Provide general encouragement  Provide contingent rewards |
| **Name**  Monster Heart Medic  **Developer**  The Lawrence Hall of Science, University of California  **Store**  iTunes  Google Play | **Cost**  Free  **User rating**  Average: 4.0  Number of user ratings: 34  **Target group**  Children  Target age range:  9-12 years  **Health behaviour**  Diet  Physical activity | **App type**  Educational  Exergame  **App features**  n= 4  Password required: no  Inbuilt accelerometer: no  GPS: no  Educational information: yes   - About cardiovascular health system, risk factors), e.g. “To lower his blood pressure Ragnar will need to make some serious changes. He needs to eat healthier foods and exercise more.”   Alignment with guidelines: yes  Social networking option: no  Push notifications: no  Reminders: no  Awards/rewards: yes   - Earn health achievements that let players build their knowledge of the cardiovascular system and healthy living.   Gamification: yes   - Educational adventure game that explores the cardiovascular system and how it is affected by healthy living. - Game character ‘monster Ragnar’ - Game guides through steps to help diagnose monster Ragnar’s health problems - Unlock information about the cardiovascular systems - Unlock stories of other characters with cardiovascular diseases - Gain coins when reaching a destination in the virtual town   **Combined with other devices**  None | **Behaviour change technique**  n = 3  Provide information about the health behaviour link  Provide information on consequences  Provide instruction |
| **Name**  Ninja Fitness  **Developer**  Heckr  **Store**  iTunes | **Cost**  Free  **User rating**  Average: 4.5  Number of user ratings: 107  **Target group**  Children  Target age range:  4+ years  **Health behaviour**  Physical activity | **App type**  Educational  Exergame  **App features**  n = 4  Password required: no  Inbuilt accelerometer: no  GPS: no  Educational information: yes   - Provides videos about how to perform exercises and also information on the benefits of exercise, zen is good for the mind)   Alignment with guidelines: no  Social networking option: yes   - Share workout achievement on Facebook or Twitter   Push notifications: no  Reminders: no  Awards/rewards: yes   - As you work through the challenges, you earn ninja points & belts.   Gamification: yes   - Ninja trainer ‘Grandmaster Old Beard’ to coach the workouts - Earning points (ninja stars) and reaching next level ‘belts’ for completing workouts - Buy weapons, outfits and armor with the points accrued   **Combination with other devices**  None | **Behaviour change techniques**  n = 8  Provide information about behaviour-health link  Provide general encouragement  Provide instruction  Provide feedback on performance  Set graded tasks  Model or demonstrate behaviour  Prompt self-monitoring of behaviour  Provide contingent rewards |
| **Name**  Kid’s Fitness  **Developer**  Fitivity  **Store**  Google Play | **Cost**  Freemium  **User rating**  Average: 4.3  Number of user ratings: 28  **Target group**  Children, adolescents  Target age range: 12+ years  n.r.  **Health behaviour**  Physical activity | **App type**  Educational  Work outs, training  **App features**  n = 2  Password required: no (only if linking to social media)  Inbuilt accelerometer: no  GPS: no  Educational information: yes   - Link to healthy recipes, weight loss information - Fitness workouts and training circuits - Video demonstrations of workouts - Workout instructions   Alignment with guidelines: no  Social networking option: yes   - Sign in to Facebook or Google to share posts - Create posts, add images or videos - Users share posts around exercise, fitness and nutrition - Create a user profile with photo   Push notifications: no  Reminders: no  Awards/rewards: no  Gamification: no  **Combination with other devices**  None | **Behaviour change techniques**  n = 6  Provide information about behaviour-health link  Provide instruction  Model or demonstrate the behaviour  Provide information about others’ approval  Set graded tasks  Provide opportunities for social comparison |
| **Name**  Playtime Kid Crono  **Developer**  RecreaVision  **Store**  iTunes | **Cost**  Paid  **User rating**  Average: 4+  Number of user ratings: 50  **Target group**  Parents and children  Target age range:  3-12 years  **Health behaviour**  Physical activity  Sedentary behaviour (decreasing screen time and increasing physical activity/play time) | **App type**  Educational  Exergame  **App features**  n = 7  Password required: yes   - The screen time timer can only be controlled by the parent through a 4-digit PIN.   Inbuilt accelerometer: no  GPS: no  Educational information: yes   - Very basic information at the beginning in regards to screen time in children and sets recommended screen time for children in accordance to their age.   Alignment with guidelines: yes   - Screen time limit of ≤ 2 hours/day   Social networking option: no  Push notifications: yes  Reminders: yes   - When screen time exceeds timer, you are sent reminders to stop screen time and go and play   Awards/rewards: yes   - Parents can grant or remove rewards to the child, i.e. coins and cups to encourage the child stop using the device once the granted time has elapsed.   Gamification: yes   - Each child has his own avatar whose mood changes according to playtime. The more playtime the more tired the avatar looks. With the agreed playtime, the avatar shows a happy face.   **Combination with other devices**  None | **Behaviour change techniques**  n = 9  Provide instruction  Provide general encouragement  Prompt intention formation  Prompt specific goal setting  Prompt self-monitoring of behaviour  Provide feedback on performance  Provide contingent rewards  Use follow-up prompts  Time management |
| **Name**  Kurbo Health Coaching – Kids & Teens Food Tracker  **Developer**  Kurbo Health, Inc.  **Store**  iTunes  Google Play | **Cost**  Paid  **User rating**  Average: 4.5  Number of user ratings:  185  **Target group**  Children, adolescents, families  Target age range:  5-18 years  **Health behaviour**  Diet  Physical activity (exercise) | **App type**  Educational  Diet and exercise tracker  Virtual coaching  **App features**  n = 7  Password required: yes  Inbuilt accelerometer: no  GPS: no  Educational information: yes   - Virtual coaching through app-delivered messages, texts, and notifications that provide personalized feedback, encouragement and recommendations - Weekly coaching session via video, telephone or text - Nutritional education information via app (e.g., games, videos)   Alignment with guidelines: yes   - Portion size control of foods - Green, yellow and red light foods   Social networking option: no  Push notifications: yes   - Notices of chat messages from personal coach, as well as tips and reminders   Reminders: yes   - Daily reminders to track food and exercise   Awards/rewards: yes   - Earn points and badges for reaching food intake goals   Gamification: yes   - Fun games that help learn about nutrition, the healthy food pyramid, earn badges.   Food and exercise tracker to record behaviour in an exercise and food diary  Progress tracker to see progress over time  **Combination with other devices**  Can be connected to Fitbit | **Behaviour change techniques**  n = 12  Prompt intention formation  Provides general encouragement  Model or demonstrate the behaviour  Prompt specific goal setting  Prompt review of behavioural goals  Prompt self-monitoring of behaviour  Provide feedback on performance  Provide instruction  Prompt practice  Provide contingent rewards  Provides opportunities for social comparison  Relapse prevention |
| **Name**  NFL Play 60  **Developer**  American Heart Association  **Store**  iTunes  Google Play | **Cost**  Free  **User rating**  Average: 4.6  Number of user ratings:  357  **Target group**  Children  Target age range:  9-11 years  **Health behaviour**  Physical activity | **App type**  Educational  Exergame  **App features**  n = 5  Password required: no  Inbuilt accelerometer: yes  GPS: no  Educational information: yes   - Getting educational tips (e.g., Dancing is a great way to burn calories and have fun at the same time, When watching TV, get up during commercial breaks and try and do as many situps or jumping jacks before the show comes back on, keep hydrated)   Alignment with guidelines: no  Social networking option: yes   - Connects to Facebook   Push notifications: no  Reminders: no  Awards/rewards: yes   - Collect coins to buy character team wear, unlock funky characters, earn stars for a chance at the prize wheel, play daily goal to unlock more rewards)   Gamification: yes   - Collect coins and stars, compare yards run to NFL players   **Combination with other devices**  None | **Behaviour change techniques**  n = 10  Prompt intention formation  Prompt specific goal setting  Prompt self-monitoring of behaviour  Provide general encouragement  Set graded tasks  Provide instruction  Provide feedback on performance  Provide contingent rewards  Provide opportunities for social comparison  Prompt practice |
| **Name**  iBitz  **Developer**  GeoPalz LLC  **Store**  iTunes  Google Play | **Cost**  Free  **User rating**  Average: 4  Number of user ratings: 128  **Target group**  Children  Target age range:  6-8years  **Health behaviour**  Physical activity  Sedentary behaviour | **App type**  Exergame  Information/education  Advice/tips/strategies/skills training  **App features**  n = 6  Password required: yes  Inbuilt accelerometer: yes  GPS: no  Educational information: yes   - Example: Do you know the average person walks 115,00 miles in a lifetime?   Alignment with guidelines: no  Social networking option: yes   - Connects to Facebook, Youtube, Twitter, Instagram   Push notifications: no  Reminders: no  Awards/rewards: yes   - Set daily and long-term goals and redeem activity for games and rewards, such as screen time, adventure or time with parent.   Gamification: yes   - As child does activity, they power their virtual character along an adventure trough the galaxy, exploring different plants.   **Combination with other devices**  Website, wearable tracker (e.g. iBitz Kids pedometer) | **Behaviour change techniques**  n = 10  Provide information about behaviour-health link  Prompt intention formation  Set graded tasks  Prompt specific goal setting  Prompt review of behavioural goals  Provide general encouragement  Provide instruction  Provide feedback on performance  Provide contingent rewards  Provide opportunities for social comparison |
| **Name**  Fruits Learning  **Developer**  Smart App Array  **Store**  Google Play | **Cost**  Free  **User rating**  Average: 4.5  Number of user ratings:  35  **Target group**  Pre-schoolers  School children  Pre-Teens  Target age range:  Not reported  **Health behaviour**  Diet | **App type**  Educational  Game  **App features**  n = 3  Password required: no  Inbuilt accelerometer: no  GPS: no  Educational information: yes   - Increase children’s recognition and knowledge of fruits   Alignment with guidelines: no  Social networking option: no  Push notifications: no  Reminders: no  Awards/rewards: yes   - Clapping and cheers via voice when a game is won - Earn points and stars for correct answers in the games   Gamification: yes   - Memory games, shadow games and knowledge tests to identify fruits   **Combination with other devices**  None | **Behaviour change techniques**  n= 3  Provide general encouragement  Provide contingent rewards  Provide feedback on performance |
| **Name**  Fruits & Veg Book  **Developer**  Negorp  **Store**  Google Play | **Cost**  Free  **User rating**  Average: 4.0  Number of user ratings:  163  **Target group**  Children  Target age range:  Not reported  **Health behaviour**  Diet | **App type**  Educational  **App features**  n = 1  Password required: no  Inbuilt accelerometer: no  GPS: no  Educational information: yes   - Visual presentation of fruits and vegetables combined with education (via text and voice recording) about the type of fruit and vegetables and its health benefits (i.e., vitamins, minerals, prevention of specific chronic diseases)   Alignment with guidelines: no  Social networking option: no  Push notifications: no  Reminders: no  Awards/rewards: no  Gamification: no  **Combination with other devices**  None | **Behaviour change techniques**  n = 0 |
| **Name**  More Salad  **Developer**  Maverick Software LLC  **Store**  iTunes  Google Play | **Cost**  Paid    **User rating**  4  **Number of user ratings**  2251  **Target group**  Children 4+ years  Adolescents  Adults  **Health behaviour**  Diet | **App type**  Educational  **App features**  n = 2  Password required: no  Inbuilt accelerometer: no  GPS: no  Educational information: no  Alignment with guidelines: no  Social networking option: yes   - Connects to Facebook, Twitter   Push notifications: no  Reminders: no  Awards/rewards: no  Gamification: yes   - Challenge your friends   **Combined with other devices**  None | **Behaviour change technique**  n = 2  Provide opportunities for social comparison  Prompt practice |
| **Name**  Plato's Health Cannon  **Developer**  By Creative Apps S.A.L  **Store**  iTunes  Google Play | **Cost**  Free  **User rating**  Average: 4.8  Number of user ratings:  28  **Target group**  Young children  Children  Age range: 4+ years  **Health behaviour**  Diet | **App type**  Educational  **App features**  n = 4  Password required: no  Inbuilt accelerometer: no  GPS: no  Educational information: no  Alignment with guidelines: yes  Social networking option: yes   - Connects to Facebook   Push notifications: no  Reminders: no  Awards/rewards: yes   - Earn coins and stars   Gamification: yes   - Aim the cannon, power up and shoot healthy food into one pod and junk into another pod.   **Combination with other devices**  None | **Behaviour change techniques**  n = 5  Provide contingent rewards  Set graded tasks  Provide instruction  Provide opportunities for social comparison  Provide feedback on performance |
| **Name**  Cookie Calls  **Developer**  By Sesame Street  **Store**  iTunes  Google Play | **Cost**  Fremium  **User rating**  Average: 4  Number of user ratings:  546  **Target group**  Young children  Age range: 5 years and under  **Health behaviour**  Diet  Physical activity | **App type**  Educational  Advice/Tips/Strategies/Skills training  **App features**  n = 4  Password required: no  Inbuilt accelerometer: no  GPS: no  Educational information: yes   - Each phone call 'Cookie Monster' gives tips depending on what pack you have purchased e.g. Eat your colours-encourages healthy eating, Healthy Habits- encourages exercise.   Alignment with guidelines: yes  Social networking option: no  Push notifications: yes   - Receive scheduled calls from the Cookie Monster   Reminders: no  Awards/rewards: no  Gamification: yes   - Receive calls from the Cookie Monster   **Combination with other devices**  None | **Behaviour change techniques**  n = 5  Provide instruction  Provide information on consequences  Provide general encouragement  Model or demonstrate the behaviour  Prompt practice |
| **Name**  Tummyfish  **Developer**  Nestle Middle East  **Store**  iTunes  Google Play | **Cost**  Free  **User rating**  Average: 4.4  Number of user ratings:  397  **Target group**  Young children  Children  Age range: 4-12 years  **Health behaviour**  Diet | **App type**  Game  Educational  Advice/Tips/Strategies/Skills training  **App features**  n = 8  Password required: yes   - Parents need to authorise app use   Inbuilt accelerometer: no  GPS: no  Educational information: yes   - In parents section, there are tips for parents- fluid requirements with the recommended daily water consumption figures, being a role model.   Alignment with guidelines: yes  Social networking option: yes   - Connects to Facebook and Instagram   Push notifications: yes   - To remind child to drink more water   Reminders: yes   - Can choose particular times in the day for reminders to the child   Awards/rewards: yes   - Drink water to earn a 'Good day' star and unlock new games and toys   Gamification: yes   - Water makes fish character in app happy and sugary drinks makes fish character sad, the more good days the child has the more their fish character grows.   **Combination with other devices**  None | **Behaviour change techniques**  n = 9  Provide information on consequences  Provide instruction  Prompt specific goal setting  Prompts self-monitoring of behaviour  Provide feedback on performance  Provide contingent rewards  Prompt identification as a role model  Time management  Prompt practice |
| **Name**  Nature Cat's Great Outdoors  **Developer**  PBS Kids  **Store**  iTunes  Google Play | **Cost**  Free  **User rating**  Average: 4.3  Number of user ratings:  108  **Target group**  Young children  Children  Age range: 8 years & under  **Health behaviour**  Physical activity | **App type**  Game  **App features**  n = 1  Password required: no  Inbuilt accelerometer: no  GPS: no  Educational information: no  Alignment with guidelines: no  Social networking option: no  Push notifications: no  Reminders: no  Awards/rewards: no  Gamification: yes   - Uses camera, microphone, sketching & art tools.   **Combination with other devices**  None | **Behaviour change techniques**  n = 4  Provide instruction  Prompt self-monitoring of behaviour  Provide general encouragement  Prompt practice |
| **Name**  GoNoodle Kids  **Developer**  GoNoodle Inc  **Store**  iTunes  Google Play | **Cost**  Free  **User rating**  Average: 4  Number of user ratings: 22  **Target group**  Young children  Children  Teens  Age range: 6-8 years  **Health behaviour**  Diet  Physical Activity  Sedentary behaviour | **App type**  Game  Educational  **App features**  n = 4  Password required: yes  Inbuilt accelerometer: no  GPS: no  Educational information: yes   - Videos with tips for physical activity, moving more, and mindfulness)   Alignment with guidelines: no  Social networking option: no  Push notifications: no  Reminders: no  Awards/rewards: yes   - After every 10 videos children watch, they earn items for their virtual character.   Gamification: yes   - Offers movement videos and games that deliver, reward, and measure physical activities of children.   **Combination with other devices**  None | **Behaviour change techniques**  n = 8  Prompt intention formation  Provide instruction  Provide general encouragement  Prompt self-monitoring of behaviour  Model or demonstrate the behaviour  Prompt practice  Provide contingent rewards  Stress management |
| **Name**  Cooking Fun for Kids  **Developer**  Playrific  **Store**  iTunes  Google Play | **Cost**  Free  **User rating**  Average: 4.5  Number of user ratings: 45  **Target group**  Young children  Target age range:  6-8years  **Health behaviour**  Diet | **App type**  Educational  **App features**  n = 2  Password required: no  Inbuilt accelerometer: no  GPS: no  Educational information: yes   - Videos, games, books, photos,recipes & puzzles designed to educate children to cook and eat healthfully   Alignment with guidelines: no  Social networking option: no  Push notifications: no  Reminders: no  Awards/rewards: no  Gamification: yes   - Puzzles, dress up and memory games, art   **Combination with other devices**  None | **Behaviour change techniques**  n = 3  Provide general encouragement  Provide instruction  Model or demonstrate the behaviour |

Abbreviations:
