# Supplementary material for: Apps to improve diet, physical activity and sedentary behaviour in children and adolescents: a review of quality, features and behaviour change techniques
Source: Int J Behav Nutr Phys Act. 2017 Jun 24;14:83. doi: 10.1186/s12966-017-0538-3 (PMC5483249; doi:10.1186/s12966-017-0538-3)
Supplement: Supplementary file 4 — MARS scores and inter-rater reliability. (DOCX 49 kb) [file 12966_2017_538_MOESM4_ESM.docx]

Additional file 4: MARS scores and inter-rater-reliability

|  | 1. Entertainment | 1. Interest | 1. Customisation | 1. Interactivity | 1. Target Group | 1. Performance | 1. Ease of Use | 1. Navigation | 1. Gestural Design | 1. Layout | 1. Graphics | 1. Visual Appeal | 1. Accuracy | 1. Goals | 1. Quality | 1. Quantity | 1. Visual | 1. Credibility | 1. Evidence Base |
| --- | --- | --- | --- | --- | --- | --- | --- | --- | --- | --- | --- | --- | --- | --- | --- | --- | --- | --- | --- |
| Walkr | 4.0 | 4.5 | 3.5 | 4 | 3 | 4 | 2 | 3 | 3.5 | 2.5 | 3.5 | 3.5 | 4.5 | 2 | 0 | 0 | 3.5 | 1 | 0 |
| Wokamon | 5 | 4.5 | 4.5 | 3 | 4 | 4.5 | 3 | 3.5 | 4 | 4 | 3.5 | 4 | 4.5 | 4.5 | 0 | 3.5 | 4.5 | 2.5 | 0 |
| Zomibies Run 5K | 4.5 | 4.5 | 3.5 | 3 | 3 | 4 | 4.5 | 4.5 | 5 | 3.5 | 2 | 3 | 4.5 | 4.5 | 4 | 5 | 4 | 1 | 0 |
| Swordkit Kids | 4 | 3.5 | 3 | 3 | 4 | 4 | 4 | 4 | 4 | 4 | 4 | 4 | 4 | 4 | 4 | 2 | 4 | 3 | 0 |
| Fitbit | 4.5 | 4.5 | 4 | 4.5 | 4.5 | 5 | 4 | 4 | 4.5 | 5 | 4.5 | 4 | 4.5 | 4.5 | 4 | 4 | 4.5 | 3 | 3 |
| Awesome Eats | 4 | 4 | 3 | 1.5 | 3 | 4.5 | 5 | 5 | 5 | 5 | 5 | 4 | 3 | 0 | 3.5 | 3 | 4.5 | 3 | 0 |
| Monster Heart Medic | 4 | 4 | 2 | 3.5 | 3.5 | 4.5 | 2.5 | 2.5 | 2.5 | 4 | 4 | 3.5 | 3.5 | 0 | 4 | 3.5 | 3.5 | 4 | 0 |
| Dungeon Runner | 2.5 | 3 | 2.5 | 3 | 2.5 | 4 | 3.5 | 3 | 4 | 2.5 | 2 | 2.5 | 3.5 | 0 | 0 | 0 | 2 | 1 | 0 |
| Ninja Fitness | 4.5 | 4.5 | 4.5 | 2.5 | 3.5 | 4.5 | 4.5 | 3.5 | 3.5 | 4.5 | 3.5 | 3.5 | 4.5 | 4.5 | 4.5 | 3.5 | 4.5 | 3 | 0 |
| Playtime Kid Crono | 2.5 | 2.5 | 4.5 | 3.5 | 4.5 | 4.5 | 3 | 3 | 4.5 | 3.5 | 3.5 | 3.5 | 4 | 3.5 | 2.5 | 0 | 0 | 2.5 | 0 |
| Cooking Fun for Kids | 4.5 | 4.5 | 1.5 | 1.5 | 5 | 4.5 | 5 | 4.5 | 5 | 4.5 | 4.5 | 4 | 5 | 0 | 4.5 | 4 | 4.5 | 3 | 0 |
| More Salad | 2.5 | 2 | 1.5 | 1.5 | 3 | 1 | 4.5 | 3 | 3 | 2 | 1.5 | 2.5 | 3 | 0 | 0 | 0 | 4 | 2 | 0 |
| Fruits Learning | 4 | 4 | 1 | 1.5 | 4.5 | 4.5 | 5 | 3.5 | 4 | 5 | 5 | 4 | 4.5 | 3.5 | 0 | 0 | 4 | 2.5 | 0 |
| Fruit & Veg Book | 2 | 2.5 | 1 | 1 | 3.5 | 4.5 | 5 | 4 | 4 | 2.5 | 3.5 | 2.5 | 4 | 0 | 4 | 2.5 | 2.5 | 1.5 | 0 |
| Kid’s Fitness | 2.5 | 3.5 | 1.5 | 1.5 | 3 | 3.5 | 3 | 3.5 | 3 | 2.5 | 3.5 | 2.5 | 4 | 0 | 3.5 | 3.5 | 4 | 1.5 | 0 |
| Runbit | 4 | 4.5 | 3 | 2.5 | 3.5 | 4 | 3.5 | 4 | 4 | 4 | 3 | 3 | 4.5 | 4 | 4 | 3.5 | 4.5 | 3 | 0 |
| NFL Play 60 | 5 | 5 | 4.5 | 4 | 5 | 4.5 | 5 | 4.5 | 4.5 | 4.5 | 5 | 5 | 4.5 | 5 | 4.5 | 4.5 | 4.5 | 4.5 | 0 |
| iBitz | 3.5 | 3.5 | 4.5 | 3.5 | 4.5 | 1 | 4.5 | 4.5 | 4 | 4.5 | 4 | 3.5 | 4.5 | 4.5 | 3 | 1 | 4.5 | 3 | 0 |
| Kurbo | 4 | 4 | 4.5 | 4.5 | 4.5 | 3.5 | 3.5 | 3.5 | 3.5 | 4 | 4 | 3.5 | 4.5 | 4.5 | 4.5 | 5 | 3 | 4 | 3 |
| Pokemon Go | 5 | 3 | 4.5 | 4.5 | 4.5 | 4 | 4.5 | 5 | 5 | 4 | 4.5 | 4.5 | 4.5 | 4.5 | 4.5 | 4.5 | 4.5 | 1 | 0 |
| Plato’s Healthy Canon | 3.5 | 3.5 | 2 | 2 | 4.5 | 3.5 | 3.5 | 3 | 4.5 | 3 | 4 | 4 | 4 | 0 | 3 | 3 | 3.5 | 2.5 | 0 |
| Cookie Calls | 4 | 4 | 3.5 | 3.5 | 4.5 | 4.5 | 5 | 5 | 5 | 5 | 4.5 | 4.5 | 5 | 3.5 | 4 | 3.5 | 4 | 3.5 | 0 |
| Tummyfish | 4.5 | 4.5 | 4.5 | 4.5 | 5 | 4.5 | 5 | 4.5 | 5 | 4.5 | 5 | 5 | 5 | 4.5 | 4.5 | 4.5 | 4.5 | 1 | 0 |
| Nature Cat’s Great Outdoors | 4.5 | 4.5 | 3 | 2 | 5 | 3.5 | 4.5 | 4 | 4 | 3.5 | 4 | 4.5 | 4.5 | 3.5 | 4 | 2 | 4.5 | 3.5 | 0 |
| GoNoodle Kids | 4.5 | 5 | 4 | 2.5 | 4.5 | 4 | 5 | 4.5 | 5 | 5 | 4.5 | 5 | 5 | 4.5 | 4.5 | 4 | 4.5 | 3.5 | 3.5 |

Additional file 2: MARS coding and interrater-reliability - continued

|  | Engagement score | Functionality score | Aesthetics score | Information quality score | Total MARS score (mean across sub-scores) |
| --- | --- | --- | --- | --- | --- |
| Walkr | 3.8 | 3.1 | 3.2 | 1.6 | 2.9 |
| Wokamon | 4.2 | 3.8 | 3.8 | 2.8 | 3.7 |
| Zombies Run 5K | 3.7 | 4.8 | 2.8 | 3.3 | 3.7 |
| Swordkit Kids | 3.5 | 4.0 | 4.0 | 3.0 | 3.6 |
| Fitbit | 4.4 | 4.4 | 4.5 | 3.9 | 4.3 |
| Awesome Eats | 3.1 | 4.9 | 4.7 | 2.4 | 3.8 |
| Mosnter Heart Medics | 3.4 | 3.0 | 3.8 | 2.6 | 3.2 |
| Dungeon Runner | 2.7 | 3.6 | 2.3 | 0.9 | 2.4 |
| Ninja Fitness | 3.9 | 4.0 | 3.8 | 3.5 | 3.8 |
| Playtime Kid Crono | 3.5 | 3.8 | 3.5 | 1.8 | 3.2 |
| Cooking Fun for Kids | 3.4 | 4.8 | 4.3 | 3.0 | 3.9 |
| More Salad | 2.1 | 4.3 | 2.0 | 1.3 | 2.4 |
| Fruits Learning | 3.0 | 4.3 | 4.7 | 2.1 | 3.5 |
| Fruit & Veg Book | 2.0 | 4.4 | 2.8 | 2.1 | 2.8 |
| Kid’s Fitness | 2.4 | 3.3 | 2.8 | 2.4 | 2.7 |
| Runbit | 3.5 | 3.9 | 3.3 | 3.4 | 3.5 |
| NFL Play 60 | 4.7 | 4.6 | 4.8 | 2.9 | 4.3 |
| iBitz | 3.9 | 3.5 | 4.0 | 2.9 | 3.6 |
| Kurbo | 4.3 | 3.5 | 3.8 | 4.1 | 3.9 |
| Pokemon Go | 4.3 | 4.6 | 4.3 | 3.4 | 4.2 |
| Plato’s Healthy Canon | 3.1 | 3.6 | 3.7 | 2.3 | 3.2 |
| Cookie Calls | 3.9 | 4.9 | 4.7 | 3.4 | 4.2 |
| Tummyfish | 4.6 | 4.8 | 4.8 | 3.4 | 4.4 |
| Nature Cat’s Great Outdoors | 3.8 | 4.0 | 4.0 | 3.1 | 3.7 |
| GoNoodle Kids | 4.1 | 4.6 | 4.8 | 4.2 | 4.4 |
| **Kalpha** |  |  |  |  | **0.73** |

Abbreviations: Kalpha = Krippendorff’s alpha
